# Cadmium‐Free Kesterite Thin‐Film Solar Cells with High Efficiency Approaching 12%

**DOI:** 10.1002/advs.202302869

**Published:** 2023-06-30

**Authors:** Nafees Ahmad, Yunhai Zhao, Fan Ye, Jun Zhao, Shuo Chen, Zhuanghao Zheng, Ping Fan, Chang Yan, Yingfen Li, Zhenghua Su, Xianghua Zhang, Guangxing Liang

**Affiliations:** ^1^ Shenzhen Key Laboratory of Advanced Thin Films and Applications Key Laboratory of Optoelectronic Devices and Systems College of Physics and Optoelectronic Engineering Shenzhen University Shenzhen 518060 P. R. China; ^2^ CNRS ISCR (Institut des Sciences Chimiques de Rennes) UMR 6226 Université de Rennes Rennes F‐35000 France; ^3^ Sustainable Energy and Environment Thrust Jiangmen Laboratory of Carbon Science and Technology The Hong Kong University of Science and Technology (Guangzhou) Guangzhou 510000 P. R. China; ^4^ College of Materials and Energy Engineering Guizhou Institute of Technology Guiyang 550003 P. R. China

**Keywords:** atomic layer deposition, buffer layers, cadmium‐free solar cells, efficiency, kesterite

## Abstract

Cadmium sulfide (CdS) buffer layer is commonly used in Kesterite Cu_2_ZnSn(S,Se)_4_ (CZTSSe) thin film solar cells. However, the toxicity of Cadmium (Cd) and perilous waste, which is generated during the deposition process (chemical bath deposition), and the narrow bandgap (≈2.4 eV) of CdS restrict its large‐scale future application. Herein, the atomic layer deposition (ALD) method is proposed to deposit zinc–tin‐oxide (ZTO) as a buffer layer in Ag‐doped CZTSSe solar cells. It is found that the ZTO buffer layer improves the band alignment at the Ag‐CZTSSe/ZTO heterojunction interface. The smaller contact potential difference of the ZTO facilitates the extraction of charge carriers and promotes carrier transport. The better p–n junction quality helps to improve the open‐circuit voltage (*V*_OC_) and fill factor (FF). Meanwhile, the wider bandgap of ZTO assists to transfer more photons to the CZTSSe absorber, and more photocarriers are generated thus improving short‐circuit current density (*J*sc). Ultimately, Ag‐CZTSSe/ZTO device with 10 nm thick ZTO layer and 5:1 (Zn:Sn) ratio, Sn/(Sn + Zn): 0.28 deliver a superior power conversion efficiency (PCE) of 11.8%. As far as it is known that 11.8% is the highest efficiency among Cd‐free kesterite thin film solar cells.

## Graphical abstract

Eco‐friendly ZTO buffer layer is demonstrated in Ag‐doped‐CZTSSe thin film solar cells. Benefiting from the high transparency, wide bandgap of ZTO, and the favorable band alignment at the ZTO/Ag‐CZTSSe heterojunction effectively suppress interface recombination and improve the photovoltaic parameters thus the champion device exhibits 11.8% efficiency, which is the highest efficiency for Cd‐free kesterite thin film solar cells.

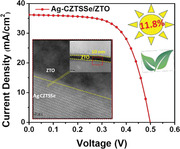

## 1 Introduction

Kesterite (CZTSSe) thin film solar cells have received enormous attention owing to its outstanding properties such as higher absorption coefficient (>10^4^ cm^−1^) in the visible spectrum, appropriate bandgap (1.0–1.5 eV), non‐toxic nature, and earth‐abundant constituent.^[^ ^1^, ^2^, ^3^ ^]^ Therefore, CZTSSe‐thin film solar cells have huge potential to replace the toxic and rare elements (Cd, In, Te, and Ga), for the sophisticated CdTe and Cu(In,Ga)Se_2_ (CIGS) absorber.^[^ ^4^, ^5^ ^]^ The typical structure of CZTSSe devices, Mo/CZTSSe/CdS/ITO/Ag, is derived from CIGS solar cells, which have been put into commercial production.^[^ ^6^ ^]^ The efficiency of CZTSSe is limited by a large number of intrinsic defects as well as undesirable band alignment, which lead to short minority carrier lifetimes and deleterious bandgap/potential fluctuations.^[^ ^7^, ^8^ ^]^ To address these problems, researchers have proposed strategies such as cation substitution, heterojunction annealing, and interface engineering to improve the device efficiency to more than 13%.^[^ ^9^, ^10^, ^11^ ^]^ However, due to the toxicity of Cd and the harmful waste produced by the chemical bath deposition (CBD) process, the development of environmentally friendly CZTSSe thin film solar cells with a Cd‐free buffer is necessary to promote their application. Besides, the CIGS cell with a world record efficiency of 23.35% currently uses Zn(O,S) buffer in place of CdS.^[^ ^12^, ^13^ ^]^ Taking into account the historical development of CIGS, it is reasonable to expect the CZTSSe solar cells without Cd buffer could achieve a highly promising PCE in comparison to CdS buffered CZTSSe solar cells.

In CZTSSe solar cells, several types of eco‐friendly and Cd‐free buffer layers including In_2_S_3_, ZnS(O,OH), (Zn Mg)O, Zn(O, S), and (Zn, Sn)O have been used.^[^ ^14^, ^15^, ^16^, ^17^, ^18^, ^19^, ^20^, ^21^, ^22^, ^23^, ^24^, ^25^, ^26^, ^27^, ^28^, ^29^, ^30^, ^31^, ^32^, ^33^, ^34^, ^35^ ^]^ Among them, (Zn, Sn)O commonly known as “ZTO” is a propitious substitute due to its non‐toxicity and promising results that have been reported previously by the ALD method. ALD technique is a highly favorable method since it is well‐suited with other vacuum‐based strategies used in CZTS/CZTSSe thin film solar cells.^[^ ^30^ ^]^ Besides, ALD allows fastidious control of stoichiometry, atomic scale uniformity, and surface homogeneity across a large area and has demonstrated its effectiveness as a buffer and passivation layer in solar cells.^[^ ^31^, ^32^ ^]^ For example Li et al. ^[^ ^33^ ^]^ deposited ZTO (50 nm thick) by ALD on the CZTSSe thin film and demonstrated that the bandgap of ZTO and conduction band offset (CBO) can be adjusted by Zinc:Tin (Zn:Sn) ratio during the ALD processing. The resulting device with ZTO exhibited a PCE of 8.6%, which was higher than the control device (8.1%) with CdS buffer layer. Ericson et al.^[^ ^30^ ^]^ used ZTO buffer layer by ALD by changing process temperatures from 105–165 °C to regulate the ZTO/CZTS band alignment. The highest V_OC_, and FF were achieved at 105 °C, and 165 °C respectively, while a superior PCE of 9.0% was demonstrated at 145 °C. Furthermore, Cui et al.^[^ ^34^ ^]^ carefully optimized the stoichiometry and film thickness of ZTO which was deposited by the ALD process. A favorable band‐alignment between ZTO and absorber was achieved with 10 nm thick‐ZTO which enabled a notable 10% increase in *V*_OC_ and a final PCE of 9.3% was demonstrated with Cd‐free solar cells. The higher efficiency was ascribed to well‐matched band alignment and decreased interfacial defects at CZTS/ZTO heterojunction. Recently, Larsen et al.^[^ ^35^ ^]^ improved the energy band alignment at CZTS/ZTO interface by optimizing annealing time. A higher PCE of 9.7% was demonstrated with Cd‐free buffer layer. These investigations encourage more efforts toward Cd‐free buffer layer options for kesterite thin film solar cells. Lee et al.^[^ ^19^ ^]^ obtained 11.2% CZTSSe solar cells using sputtered ZTO films with fine‐tuning CBO between absorber and buffer by changing deposition temperature and the Sn/(Zn + Sn) ratio. This is the highest efficiency available for ZTO‐based buffer layers, yet still lower than CdS, and the sputtering process is more likely to damage the absorber surface compared to the atomic layer deposition method. These studies encourage us to make more efforts on ALD‐ZTO for kesterite thin‐film solar cells to improve the PCE of next generation of environmentally friendly kesterite solar cells.

**Figure 1:**
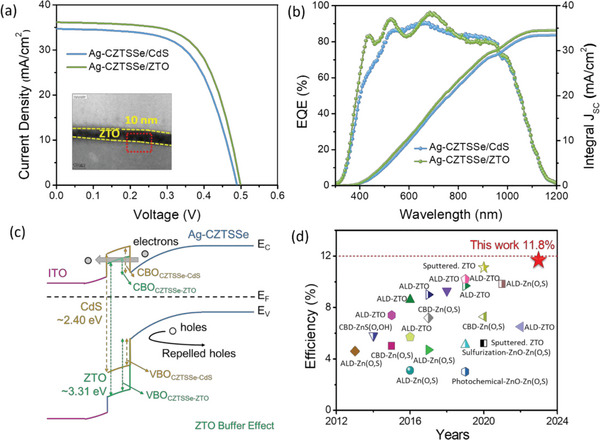
a) *J–V* curve of ZTO and CdS‐based Ag‐CZTSSe solar cells. b) EQE spectra of the corresponding devices. c) Schematic band diagrams of Ag‐CZTSSe/ZTO heterojucntion interface. d) Comparisons of PCE of Cd‐free buffer layer and champion efficiency of this work in kesterite thin film solar cells.

In this contribution, we investigated and demonstrated the role of ZTO buffer layers with different compositions and film thicknesses prepared by the ALD process in kesterite solar cells. It is widely acknowledged that Ag substitution has a positive effect on device efficiency enhancement in kesterite,^[^ ^9^, ^36^, ^37^ ^]^ and we used Ag‐substituted CZTSSe (Ag‐CZTSSe) as absorber layer material in the present work. After systematic exploration of the conditions in the Ag‐CZTSSe solar cells, the optimized device with 10 nm thickness of ZTO layer in the Zn:Sn ratio of 5:1 and Sn/(Sn + Zn) pulse ratio of ≈0.28 exhibited a superior efficiency of 11.8% compared to 10.7% of control device having CdS buffer layer in Ag‐CZTSSe solar cell (**Figure** **1**a). The smaller contact potential difference (CPD) of ZTO buffer layer is favorable to the extraction and collection of charge carriers and promotes carrier transport. In addition, the wider bandgap of ZTO facilitates to transfer greater number of photons to the CZTSSe absorber, and more photocarriers are generated in the photo‐absorber in that spectral range, thus increasing *J*sc (Figure 1b). Meanwhile, the ZTO buffer layer can optimize the band alignment of the heterojunction, with smaller CBO favoring electron transport and larger valence band offset (VBO) favoring the formation of hole barriers and reducing heterojunction interface recombination, which contributes to the V_OC_ and FF (Figure 1c). To the best of our knowledge, 11.8% is the highest PCE for using a Cd‐free buffer layer in a Cd‐free kesterite solar cell (Figure 1d and **Table** **1**). We believe that this advancement helps to drive the development of ZTO in a green environmentally friendly Cd‐free buffer layer.

**Table 1:** Photovoltaic parameters of Cd‐free buffer layers in kesterite thin film solar cells

**Absorber layer**	**Cd‐free buffer layer**	**Voc [V]**	**Jsc [mA cm^−2^]**	**FF [%]**	**PCE [%]**	**References**
CZTS	Supttered‐ZTO	0.721	14.0	51.1	5.2	Grenet et al.^[^ ^38^ ^]^
CZTS	ALD‐ZTO	0.630	18.7	49.0	5.7	Tajima et al.^[^ ^18^ ^]^
CZTS	ALD‐ZTO	0.653	16.1	61.9	6.5	Martinet al.^[^ ^32^ ^]^
CZTS	ALD‐ZTO	0.682	17.9	0.60	7.4	Platzer et al.^[^ ^17^ ^]^
CZTSSe	ALD‐ZTO	0.414	34.1	60.8	8.6	Li et al.^[^ ^33^ ^]^
CZTS	ALD‐ZTO	0.679	21.6	61.4	9.0	Ericson et al^[^ ^30^ ^]^
CZTS	ALD‐ZTO	0.720	20.5	63.5	9.3	Cui et al.^[^ ^34^ ^]^
CZTS	ALD‐ZTO	0.746	19.1	68.0	9.7	Larsen et al.^[^ ^35^ ^]^
CZTS	ALD‐ZTO	0.736	22.0	0.66	10.2	Cui et al.^[^ ^39^ ^]^
CZTSSe	Supttered‐ZTO	0.445	36.3	69.0	11.2	Lee et al.^[^ ^19^ ^]^
CZTS	Photochemical‐ZTO	0.516	16.8	35.0	3.0	Zhang et al.^[^ ^26^ ^]^
CZTSSe	ALD‐Zn(O,S)	0.313	29.4	33.0	3.1	Hong et al.^[^ ^23^ ^]^
CZTS	ALD‐Zn(O,S)	0.482	17.2	0.56	4.6	Ericson et al^[^ ^25^ ^]^
CZTSSe	ALD‐Zn(O,S)	0.327	26.6	51.1	4.7	Hong et al.^[^ ^24^ ^]^
CZTSSe	CBD‐Zn(O,S)	0.336	25.0	51.0	5.0	Steirer et al.^[^ ^28^ ^]^
CZTSe	Sulfurization‐ZnO‐Zn(O,S)	0.610	21.1	40.0	5.1	Yang et al.^[^ ^27^ ^]^
CZTSSe	CBD‐ZnS(O,OH)	0.389	29.0	52.0	5.8	Grenet et al. ^[^ ^20^ ^]^
CZTSSe	CBD‐Zn(O,S)	0.708	19.2	54.0	7.2	Huang et al.^[^ ^22^ ^]^
CZTSSe	CBD‐Zn(O,S)	0.358	33.5	60.0	7.2	Li et al.^[^ ^21^ ^]^
CZTSSe	ALD‐Zn(O,S)	0.496	35.6	56.0	9.8	Jeong et al.^[^ ^29^ ^]^
Ag‐CZTSSe	ZTO	0.498	36.28	66.53	11.8	This work

## 2 Results and Discussion

**Figure 2:**
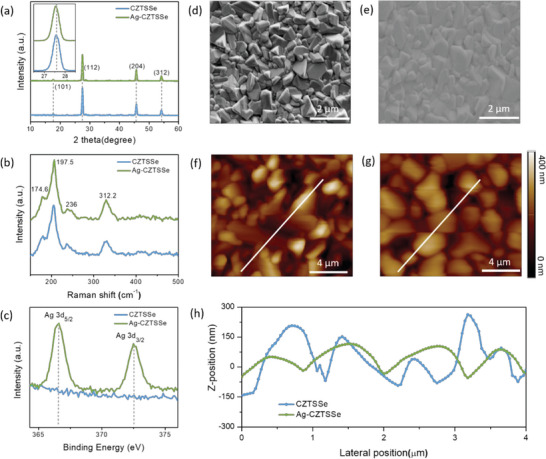
a) XRD pattern of CZTSSe and Ag‐CZTSSe films. Inset: enlarged view of the (112) lattice plane. b) Raman spectra; c) XPS spectra of Ag 3d peak of Ag‐CZTSSe film. d,e) Surface of CZTSSe and Ag‐CZTSSe thin film. f,g) AFM surface topograph images; h) surface topography line scane of CZTSSe and Ag‐CZTSSe.

First, we fabricated the CZTSSe and Ag‐doped CZTSSe absorber layer using Mo‐coated glass substrate according to the reported method in the literature.^[^ ^40^, ^41^ ^]^ Numerous studies conducted on CZTSSe have demonstrated that Ag substitution is an effective way to increase grain size and improve device performance. We have experimentally determined that a 6% Ag substitution [Ag/(Ag+Cu) = 6%] works best.^[^ ^9^ ^]^ We systematically conducted the structural and morphological analysis of CZTSSe and Ag‐CZTSSe films. **Figure** **2**a shows the X‐ray diffraction (XRD) pattern of CZTSSe and Ag‐CZTSSe films. The prominent diffraction peaks are in good agreement with the single kesterite phase without any secondary phase and impurities.^[^ ^9^ ^]^ The diffraction peaks show sharp and strong characteristics where the full width at half maximum (FWHM) values of the (112) plane reduced from 0.328 (CZTSSe) to 0.315 (Ag‐CZTSSe) due to enhanced crystallinity and enlarge crystallite size after Ag incorporation (the inset of Figure 2a). Afterward, we examined the composition and material phase of CZTSSe and Ag‐CZTSSe thin film by Raman spectroscopy. As illustrated in Figure 2b, all the prominent vibrations at 174.6, 197.5, 236, and 331.2 cm^−1^ are the distinctive modes of CZTSSe and no impurity phases are observed.^[^ ^42^ ^]^ A slightly lower peak in the Raman spectra at 236 cm^−1^ in Ag‐CZTSSe could be the result of a reduced defect after Ag incorporation.^[^ ^43^, ^44^ ^]^ X‐ray photoelectron spectroscopy (XPS) was employed to examine the oxidation states in the control and Ag‐CZTSSe film. As anticipated, the notable Ag 3d peaks of Ag‐CZTSSe thin film observe at 366.9 eV (3d_5/2_) and 372.9 eV (3d_3/2_), revealing that Ag ions have been successfully incorporated into the CZTSSe lattice (Figure 2c).^[^ ^9^ ^]^ There is no evident peak shifting for Cu 2p, Zn 2p, or Sn 3d upon Ag‐doping. To be precise, the binding energy for Cu 2p_3/2_, Cu 2p_1/2_, Zn 2p_3/2_, Zn 2p_1/2_, Sn 3d_5/2_, Sn 3d_3/2_, are 931.9, 951.8, 1022.8, 1045.4, 487.1, and 494.7 eV respectively (Figure S1, Supporting Information). Furthermore, the surface morphology of CZTSSe and Ag‐CZTSSe films was examined by scanning electron microscopy (SEM) as depicted in Figure 2d,e. Compared to CZTSSe film, the surface of Ag‐doped CZTSSe is found to be more compact, uniform, and dense. Unlike CZTSSe, the grain size of Ag‐CZTSSe film was larger without delamination or fine grains with the least voids. Figure 2f,g shows 2D AFM images of CZTSSe and Ag‐CZTSSe thin films respectively. It can also be seen surely that Ag‐CZTSSe film is compact, regular, and uniform with fewer voids which is consistent with the SEM result. Additionally, the surface roughness distinctly reduced after silver‐doping (Figure 2h). This kind of surface morphology is beneficial to achieve favorable CZTSSe surface and absorber/buffer layer interface with passivated defects.^[^ ^45^ ^]^

Then, CdS and ZTO buffer layers were fabricated by the CBD and ALD methods respectively. Temperature for deposition of ZTO buffer layers was kept fixed at 120 °C as previously used for the ZTO film ^[^ ^33^ ^]^ and different pulse ratios of Zn: Sn (3:1, 4:1, 5:1, 6:1) ALD cycles of 12, 25, 50, and 75 were used for all Zn/Sn ratios (Figure S2, Supporting Information). The growth rate was ≈0.07 nm cycle^−1^ in our experiment (see detail in the Experimental Section).

**Figure 3:**
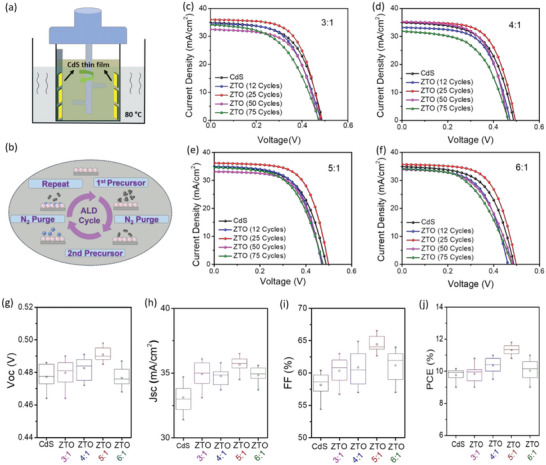
a) Schematic illustration of CdS buffer layer deposited via CBD method. b) Atomic layer deposition (ALD) process for ZTO buffer layer deposition. c–f) *J–V* curves of Ag‐CZTSSe thin film solar cell with CdS and ZTO buffer layer with Zn:Sn ratio of 3:1, 4:1, 5:1, and 6:1. The statistical distribution of photovoltaic parameters including g) V_OC_, h) J_SC_, i) FF, and j) PCE of CdS and ZTO based devices with different (Zn:Sn) ratios and 10 nm thick ZTO buffer layer.

To explore the role of ALD‐ZTO as a buffer layer in Ag‐CZTSSe device, Current density versus voltage (*J–V*) measurements were conducted for both ZTO and CdS‐based devices (reference devices). We chose four ratios of Zn: Sn (3:1, 4:1, 5:1, 6:1) with ALD cycles of 12, 25, 50, and 75. **Figure** **3**a,b illustrates the CBD process and ALD for CdS and ZTO respectively. *J–V* results with different ratios (3:1, 4:1, 5:1, 6:1) and ALD cycles (12, 25, 50, 75 cycles) are displayed in Figure 3c–f and PV parameters are listed in Table S1 (Supporting Information). Reference device with CdS delivered a *V*_OC_ of 0.490 V, *J*sc of 34.83 mA cm^−2^, FF of 62.96%, and PCE of 10.7%. Interestingly, we noted that the devices with 25 ALD cycles exhibited slightly better *V*oc, *J*sc, FF, and PCE compared to 12, 50, and 75 cycles in all series (Zn/Sn ratio; 3:1, 4:1, 6:1) as can be seen in Table S1 (Supporting Information). A champion device with a 5:1 Zn/Sn ratio, (Sn/Sn+Zn:0.28) and 10 nm thickness (25 cycles) of ZTO achieved a *V*_OC_ of 0.498 V, *J*sc of 36.28 mA cm^−2^, FF of 66.53%, and final PCE of 11.8%. This reflects that if the composition and thickness of ZTO are properly tuned, effective band alignment could be realized. Furthermore, the statistical photovoltaic performance is presented in Figure 3g–j. *J–V* results of the best devices with reference CdS and ALD‐ZTO buffer layer are listed in **Table** **2**. Up to now, 11.8% is the highest PCE obtained by Cd‐free ALD‐ZTO buffer layer. EQE shows a higher optical response from the wavelength ranges of 400–500 and 700–750 nm and can be ascribed to the wider bandgap and higher transparent nature of ZTO thin film that leads to higher *J*sc in both devices based on Ag‐CZTSSe/ZTO (Figure 1b). The Integrated *J*sc values obtained from EQE spectra were 35.16, 33.52 mA cm^−2^ which are close to the *J*sc value obtained from *J–V* measurements. We can also observe small absorption loss in the wavelength range of 550–600 nm, which can be ascribed to the interference effect of the incident light as previously reported.^[^ ^34^ ^]^ In addition, to confirm the wide applicability of ZTO, we did the same experiments on the CZTSSe absorber layer without Ag‐doping. The *J–V* curve and the EQE curve are shown in Figure S3 (Supporting Information). A reference device with CZTSSe absorber and CdS buffer layer showed a *V*_OC_ of 0.480 V, *J*_SC_ of 33.69 mA cm^−2^, FF of 61.4%, and PCE reached 10.0%. In contrast, ZTO‐based devices (5:1 Zn/Sn ratio, Sn/Sn+Zn:0.28 and 25 ALD cycles) with CZTSSe exhibited higher *V*_OC_ of 0.494 V, *J*sc of 35.10 mA cm^−2^, FF of 64.1%, and PCE of 10.9%. Furthermore, the statistical distribution of device parameters such as *V*_OC_, *J*_SC_, FF, and PCE of CZTSSe and Ag‐CZTSSe solar cells are shown in Figure S4 (Supporting Information), reflecting the reproducibility and reliability of ZTO.

**Table 2:** Photovoltaic parameters of CZTSSe and Ag‐CZTSSe thin film solar cells

**Absorber**	**ETL**	**Sn/Sn+Zn**	**Thickness [nm]**	**Voc [V]**	**Jsc [mA cm^−2^]**	**FF [%]**	**PCE [%]**
	CdS	‐	40	0.480	33.69	61.49	10.0
CZTSSe	ZTO	0.28	10	0.494	35.10	64.13	10.9
	CdS	‐	40	0.490	34.83	62.96	10.7
Ag‐CZTSSe	ZTO	0.28	10	0.498	36.28	66.53	11.8

To further explore the improved performance of ZTO‐based devices, we measured dark *J–V* for CdS and ZTO‐based devices as shown in Figure S5a,b (Supporting Information). Compared to CdS‐based devices, the devices with ZTO buffer layer showed better rectification characteristics indicating suppressed leakage current density. Moreover, we investigated diode performance parameters such as shunt conductance (*G*), series resistance (*R*), diode ideality factor (*A*), and reverse saturation current density (*J*_0_) by Equation (1).

(1) $$J\;=J_{0}\;\exp\left[\frac{q}{AkT}\left(V-RJ\right)\right]+GV-J_{L}$$

Where *J*_0_ is reverse saturation current density, *A* denotes the diode ideality factor, *R* represents series resistance, *G* stands for shunt conductance, and *J*_L_ stands for the light current density. ^[^ ^46^, ^47^, ^48^ ^]^ Figure S5c,d (Supporting Information) exhibits the plots of dJ/dV versus *V*, in order to obtain the *G* values, a tangent was drawn to the flat region under bias at the point where it intersects the Y‐axis. The *G* value was calculated as 1.3 and 0.8 mS cm^−2^ for CdS and ZTO buffer layers in the CZTSSe device, respectively. Compared to CZTSSe, Ag‐CZTSSe thin‐film solar cells showed lower *G* values of 0.9 and 0.5 mS cm^−2^ for CdS and ZTO buffer layers, respectively. The series resistance R was obtained from the plot of dV/dJ versus (J + Jsc)^−1^ extrapolating to the Y‐axis, while the ideality factor (*A*) was calculated from the slope of AkT/q. The series resistance was determined to be 2.10, 1.22, for CdS and ZTO in CZTSSe respectively. In the Ag‐CZTSSe device, the *R* values for CdS and ZTO were 1.67, and 0.97 respectively. The ideality factor was calculated to be 2.58, 1.89 for CdS and ZTO in CZTSSe, whereas the lower values of 2.11, and 1.16 were obtained for CdS and ZTO buffer layer in Ag‐CZTSSe solar cells respectively (Figure S5e,f, Supporting Information). The lower values of R and A for the ZTO layer than CdS in both (CZTSSe, and Ag‐CZTSSe) devices reflect suppressed interfacial recombination losses. Furthermore, defect state passivation of corresponding devices was analyzed by SCLC model. Figure S5g,h (Supporting Information) displays the dark *J–V* curves of CdS and ZTO‐based solar cells. Generally, the curve can be split into three (3) regions based on the value of exponent (*n*); the ohmic part (*n* = 1, at lower voltage), trap‐filled limit (TFL) region (*n* > 3, at medium voltage,), and the trap‐free child region (*n* > 2, at high voltage). When the *V* exceeds the threshold level in TFL area, the current promptly boosts, suggesting that the injected carriers have occupied the trap states. The trap‐filled limited voltages (*V*_TFL_) for CdS and ZTO‐based solar cells were calculated to be 0.26 and 0.23 V respectively. Trap state density (*N*_trap_) was obtained by Equation (2).

(2) $$N_{\mathrm{trap}}=\frac{2\varepsilon \varepsilon_{0}V_{\mathrm{TFL}}}{qL^{2}}$$

Where *ε* denotes vacuum permittivity, *ε*o denotes relative permittivity (i.e., 8 for CZTSSe), *L* is film thickness of absorber, and *q* represents the elementary charge. The ZTO‐based device exhibited a slightly lower trap state density(1.44 × 10^14^ cm^−3^) than that of the control device (1.59 × 10^14^ cm^−3^) which is slightly better quality of the active layer as shown in SEM images.

**Figure 4:**
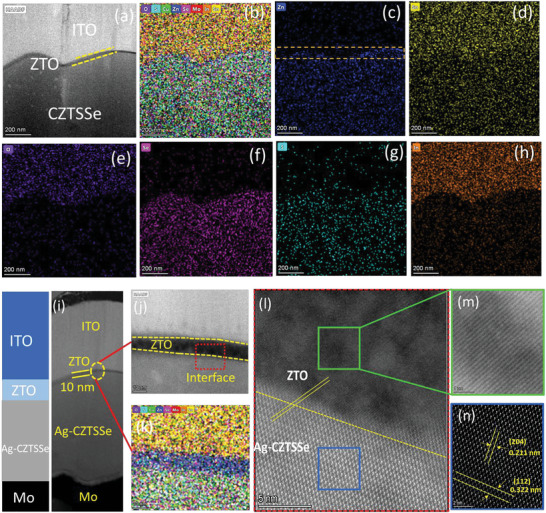
a–c) TEM‐EDS elemental line scan profiles of the interface. Elemental distribution of c) Zn, d) Sn, e) O, f) Se, g) S, h) In. i) Ag‐CZTSSe/ZTO interface. j–l) High resolution‐TEM images of cross‐section of kesterite solar cell with ZTO buffer layer. m,n) HR‐TEM images of the Ag‐CZTSSe/ZTO heterojunction interfaces.

High‐resolution transmission electron microscopy (HRTEM) was also employed on the champion device with ZTO buffer layer in order to re‐verify the microstructure at the interface of Ag‐CZTSSe/ALD‐ZTO heterojunction. **Figure** **4**a shows TEM‐EDS elemental line scan profiles of interface while the elemental distribution is exhibited in Figure 4b–h. Zn‐rich and Sn‐poor interface layers are noticeably visible in the TEM elemental mapping. This thin layer is mainly composed of Zn, Sn, and O without any obvious impurities. To investigate the detailed nanoscale elemental distribution of CZTSSe/ZTO interface, we restrained the region of concern to extra‐high magnification. As can be seen from Figure 4i–k, a thin film (thickness ≈10 nm) can be clearly distinguished as a ZTO layer which further confirms the reliability of the ALD cycle. Figure 4l reveals the lattice‐matching characteristic of Ag‐CZTSSe/ZTO interface without any discontinuity, corresponding to the compact and smooth feature. It has been reported that such a beneficial interface is conducive to alleviating the impediment of current leakage and interfacial carrier recombination.^[^ ^11^, ^49^ ^]^ Figure 4m,n shows the detailed nanoscale structure images of the Ag‐CZTSSe absorber and ZTO thin film.

In kesterite solar, suitable band alignment of the absorber with buffer layer is a prerequisite for efficient charge transport and extraction. Next, we examined the energy‐level alignment of Ag‐CZTSSe, CdS, and ZTO buffer layers by UPS measurement. It has been investigated that if the conduction band offset (CBO) is negative (CB of the buffer layer < the CB of the absorber) which results in a “cliff type” band alignment that upsurges the carrier recombination rate at CZTSSe/buffer layer interface, thus lower *V*oc of the resulting solar cells.^[^ ^19^, ^33^ ^]^ Conversely, a more positive CBO (>0.3 eV) leads to “spike type” alignment that blocks the photocurrent and causes lower *J*_SC_ and PCE. So, the favorable CBO is determined to be marginally positive or nearly zero (0–0.3 eV).^[^ ^19^, ^33^ ^]^ In this work, the bandgap (*E*_g_) of Ag‐CZTSSe, CdS, was obtained from EQE data while E_g_ of ZTO was obtained from transmittance spectra (Figure S6, Supporting Information) while the CBO was obtained from UPS data analysis (Figure S7, Supporting Information). *E*_g_, *E*_V_, and *E*_C_ of the Ag‐CZTSSe were calculated as 1.08, −5.03, and −3.95, respectively. For CdS buffer layer, the calculated values of *E*_V_, *E*_C_, and *E*_g_ were −6.19, −3.76, and 2.43 eV. On the hand, *E*_V_, *E*_C_, and *E*_g_ of ZTO were calculated as, −7.11, −3.80, and 3.31 eV. It can be seen that the CBO between Ag‐CZTSSe/CdS is 0.19, which is slightly larger than the CBO = 0.15 obtained from Ag‐CZTSSe/ZTO. Compared with the sample using CdS as the buffer layer, the device using ZTO as the buffer layer has a smaller CBO and a larger VBO value. A large CBO creates a large potential barrier, and a large potential barrier makes it difficult for photogenerated electrons to cross the p–n junction. The increase in electron collection resistance also leads to an increase of *R*_S_, which reduces the FF. Tuning the CBO to a small positive value leads to a high *V*_OC_. On the other hand, the VBO present between the buffer layer and absorber allows the hole to flow back and reduces the interface tunneling recombination. The smaller CBO of ZTO device facilitates electron transport from Ag‐CZTSSe to the buffer layer, while the larger VBO is able to form a more effective hole barrier to repel holes, thus improving carrier transport and suppressing interface carrier recombination (Figure 1c). The favorable energy band alignment at the interface of ZTO/Ag‐CZTSSe and the wider bandgap (3.31 eV) of ZTO leads to higher FF and J_SC_ in the resulting device, therefore obtaining higher performance than the CdS‐based device.

**Figure 5:**
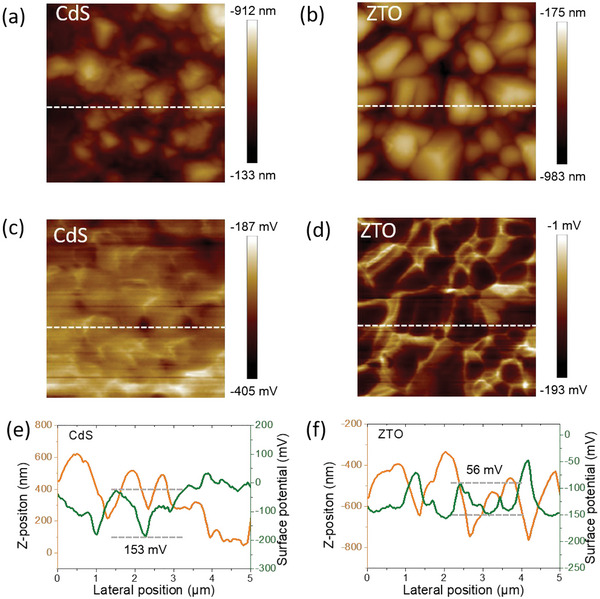
AFM scanning surface topography of a) CdS, b) ZTO. KPFM potential maps of c) CdS and d) ZTO. Topography and potential line scans of e) CdS, f) ZTO.

To explore the interface carrier recombination of the resulting device, we employed electrical impedance spectroscopy (EIS). Figure S8 (Supporting Information) illustrates the Nyquist plot of CdS and ZTO‐based devices, and the inset presents the equivalent circuit model, where *C*_rec_, *R*_s_, *R*_rec_ is the chemical capacitance, series resistance, and recombination resistance respectively. *R*_s_ corresponds to the interfacial and active layer resistance while the diameter of the arc corresponds to recombination resistance (*R*_rec_) which is indirectly proportional to the carrier recombination at the interface. Compared to the control device, slightly higher *R*_rec_ and lower *R*_s_ values of the ZTO‐based device indicate the suppressed charge recombination at the interface which is one of the reasons for higher FF in the ZTO‐based device. Kelvin probe microscopy (KPFM) was employed to analyze the surface electrical properties of buffer layers (CdS and ZTO). **Figure** **5**a–d displays the AFM micrograph and the contact potential differences (*V*_CPD_) of the two samples respectively. In the CdS sample (Figure 5e), *V*_CPD_ (153 mV) was higher than ZTO samples (56 mV) at grain boundaries which impeded carrier transport across the horizontal direction. In addition, low *V*_CPD_ at the grain surface in ZTO sample is attributed to a small potential difference which is beneficial for charge transport and extraction.^[^ ^49^ ^]^

**Figure 6:**
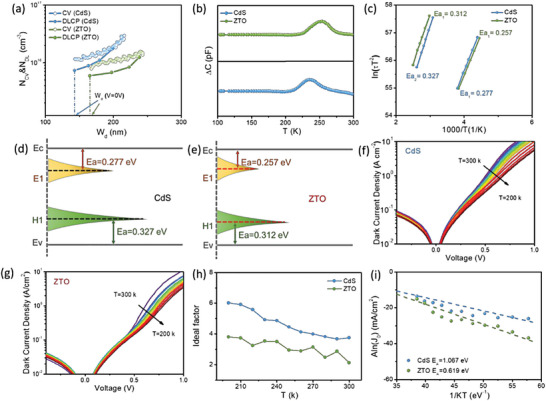
a) *C–V* and DLCP profiles, b) DLTS spectra, and c) Arrhenius plots of CdS and ZTO devices. d,e) Schematic diagram of defects in CdS and ZTO‐based devices. *J–V*–*T* plots of f) CdS device and g) ZTO device. h) *R*_S_–*T* curves and i) Aln(*J*_0_) versus 1/*kT* plot of CdS, and ZTO‐based devices.

The capacitance–voltage (*C–V*) and drive‐level capacitance profiling (DLCP) were recorded for CdS and ZTO‐based CZTSSe/Ag‐CZTSSe solar cells as illustrated in **Figure** **6**a and the findings are presented in **Table** **3**. *N*_CV_ comprises defects (bulk defects, interfacial defects) and free carriers whereas *N*_DLCP_ shows the free carriers and bulk defects. Thus, the interface defect density can be obtained from the difference between *N*_CV_ and *N*_DLCP_ (*N*_CV_ − *N*_DLCP_) at zero voltage. The plot of N_CV_ and N_DLCP_ is obtained from Equation 2. ^[^ ^40^ ^]^

(3) $$\left\{N_{C-V}=\frac{-2\varepsilon_{r,n}N_{D}}{\begin{array}{c} \left(\frac{d\left(1/C^{2}\right)}{dV}\right)qA^{2}\varepsilon_{0}\varepsilon_{r,n}\varepsilon_{r,p}N_{D}+2\varepsilon_{r,p}\\ N_{\mathrm{DLCP}}=-\frac{C_{0}^{3}}{2q\varepsilon_{0}\varepsilon_{r,p}A^{2}C_{1}}\\ x=\varepsilon_{0}\varepsilon_{r,p}A/C_{0} \end{array}}\right.$$

**Table 3:** Summary of the results derived from *C–V* and DLCP measurements for Ag‐CZTSSe/CdS and Ag‐CZTSSe/ZTO devices

Device	*N*_CV_ [cm^−3^]	*N*_DL_ [cm^−3^]		Depletion width [nm]	Interface state response
Ag‐CZTSSe/CdS	1.12 × 10^16^	7.36 × 10^15^	143		3.84 × 10^15^
Ag‐CZTSSe/ZTO	7.59 × 10^15^	5.89 × 10^15^	167		1.70 × 10^15^

Herein, *A*, *ε*_0_, *ε*_r,n_, *ε*_r,p_, and *N*_D_ is the device area, the permittivity of free space, the relative permittivity, and carrier concentration respectively. *C*_0_, and *C*_1_ are quadratic fitting parameters obtained from *C–V* data.^[^ ^50^, ^51^ ^]^ The concentration of interfacial defects density (*N*_i_) of CdS and ZTO‐based CZTSSe devices is 3.84 × 10^15^ and 1.70 × 10^15^ cm^−3^ respectively, which is attributed to optimized CZTSSe surface morphology and better ZTO/Ag‐CZTSSe heterojunction thus suppressed charge recombination. Moreover, the depletion width (*W*_d_) is a key parameter that affects the solar cell's performance. Typically, thin‐film solar cells require larger widths for efficient charge separation. In our case, *W*_d_ increased from 143 nm (CdS) to 167 nm (ZTO) in Ag‐CZTSSe thin film solar cells which is favorable for better carrier transport and reduces the interfacial recombination. The inhomogeneous static charge density generated by ionization deep defects decreases the *W*_d_, while the Ag‐CZTSSe/ZTO device undergoing the low‐temperature annealing process achieves self‐passivation of defects and thus has a lower deep level defect density than Ag‐CZTSSe/CdS devices. We believe that the decrease in deep level defect density is one of the main reasons for the increase in the *W*_d_ of Ag‐CZTSSe/ZTO devices.

To investigate the defect properties of bulk Ag‐CZTSSe with CdS and ZTO buffer layer, deep‐level transient (DLTS) spectroscopy was used. The test was conducted across the range of temperature from 90 to 330 K under reverse bias (*V*_R_ = 0.3 V), pulse voltage (*V*_P_ = 0.2 V), and pulse width of 10 ms for both devices. As shown in Figure 6b, both devices exhibit two peaks that can be attributed to donor defects (*E*1) and acceptor defects (*H*1).^[^ ^9^ ^]^ The activation energy (*E*_a_) of the defects and trap density (*N*_T_) are obtained from the Arrhenius plot as shown in Figure 6c and summarized in **Table** **4**. It has been reported that the *E*_a_ values of 0.221–0.290 eV, and 0.310–0.335 eV can be assigned to Cu_Zn_ + Sn_Zn_ defect clusters and Cu_Sn_ antisite acceptors defects respectively. ^[^ ^9^, ^40^ ^]^ In both devices, *E*_a_ values of 0.257 and 0.277 eV correspond to Cu_Zn_ + Sn_Zn_ defect clusters while *E*_a_ values of 0.312 and 0.327 eV can be assigned to Cu_Sn_ antisite acceptors defects. For Cu_Zn_ + Sn_Zn_ defect clusters, N_T_ of CdS and ZTO‐based devices was 1.11 × 10^14^ and 1.07 × 10^14^ respectively. On the other hand, the concentration of Cu_Sn_ antisite acceptor defects was determined to be 2.14 × 10^14^ and 1.99 × 10^14^ for CdS and ZTO‐based solar cells respectively. Both the defect density and activation energy of Ag‐CZTSSe/ZTO device are slightly lower than that of the control device. Schematic diagrams of defects in CdS and ZTO‐based devices are presented in Figure 6d,e. The presence of a relatively lower defect concentration (Cu_Sn_, Cu_Zn_+Sn_Zn_) within the bulk of the ZTO‐based device may be due to the environment during ALD deposition creating conditions for low‐temperature annealing of the absorber, which has been shown in the literature to promote defect passivation in the absorbent layer.^[^ ^52^, ^53^, ^54^ ^]^

**Table 4:** Summary of defects levels in Ag‐CZTSSe‐thin film solar cells obtained from DLTS spectra

Device	Defects	E_A_ [eV]	N_T_	Defect type	Possible defect
Ag‐CZTSSe/CdS	E1	0.277	1.11 × 10^14^	Donor	[Cu_Zn_+ Sn_Zn_]
Ag‐CZTSSe/CdS	H1	0.327	2.14 × 10^14^	Acceptor	Cu_Sn_
Ag‐CZTSSe/ZTO	E1	0.257	1.07 × 10^14^	Donor	[Cu_Zn_+ Sn_Zn_]
Ag‐CZTSSe/ZTO	H1	0.312	1.99 × 10^14^	Acceptor	Cu_Sn_

Temperature‐dependent current density voltage (*J–V*–*T*) analysis is the most effective technique to uncover the major recombination paths in the device.^[^ ^55^, ^56^, ^57^ ^]^ To clarify how various buffer layers affect the recombination mechanism, dark *J–V*–*T* analysis was performed at 200–300 K for Ag‐CZTSSe/CdS and Ag‐CZTSSe/ZTO samples (Figure 6f,g). The calculated results show that the ideal factor (*A*) values are larger in the CdS‐based device and strongly correlate with temperature (Figure 6h). This indicates the dominance of tunneling‐enhanced recombination, which is considered to be unfavorable in solar cells. This suggests that the presence of ZTO layer improves the junction quality and suppresses the recombination. The main recombination paths can be obtained by plotting *AlnJ*_0_ versus 1/*kT* using Equation (4).

(4) $$Aln\;J_{0}=\;Aln\left(J_{00}\right)-\frac{E_{A}}{kT}$$

where *A* is the ideal factor, *J*_0_ stands for the reverse saturation current density, *J*_00_ is the prefactor dependent on the recombination path, *E*_A,_ *k*, and *T* stand for the recombination activation energy, Boltzmann constant, and temperature respectively. According to the equation, *E*_A_ can be obtained from the sloping plot of *AlnJ*_0_ versus 1/*kT*. On comparing the *E*_A_ value to the bandgap of Ag‐CZTSSe absorber, the primary recombination route can be calculated. Figure 6i shows the *AlnJ*_0_ versus 1/*kT* plots for both devices acquired from the dark *J–V*–*T* matrics. By comparing the *E*_A_ value with the *E*_g_ of the device, the main recombination path can be determined. *E*_A_ close to *E*_g_ demonstrates that bulk recombination is dominant; while *E*_A_ smaller than *E*_g_ means interface recombination is dominant. Therefore, the difference between *E*_A_ and *E*_g_ could determine the interface recombination degree. As shown in Figure 6i, *E*_A_ value for the Ag‐CZTSSe/CdS device is 0.619 eV, which is significantly smaller than the bandgap, signifying the existence of a major recombination pathway at the Ag‐CZTSSe/CdS junction interface. *E*_A_ value for Ag‐CZTSSe/ZTO sample is 1.067 eV, which is closer to the *E*_g_ of Ag‐CZTSSe absorber, indicating that the bulk recombination is stronger than the interface recombination. This result demonstrated that the interface recombination was significantly passivated in Ag‐CZTSSe/CdS device. These findings propose that the ZTO buffer layer significantly reduces the occurrence of interface recombination.

## 3 Conclusion

In this work, we fabricated Ag‐CZTSSe thin‐film solar cells using eco‐friendly ZTO as a buffer layer deposited by ALD method with optimal stoichiometry and film thickness, which significantly improved the efficiency of kesterite solar cells. The experimental analysis confirms that the best PCE can be achieved with a 10 nm thick ZTO layer and ALD pulse ratio of 5:1 (Zn:Sn) or (Sn/Zn+Sn:0.28). The UPS data authenticate that the ZTO buffer layer can optimize the band alignment at the heterojunction, promote electron transport and form a more favorable hole barrier. The CV‐DLCP analysis and the temperature‐dependent dark *J–V* show that the interface defect density and interface recombination activation energy at the heterojunction are significantly reduced. Meanwhile, the inherent wide bandgap of ZTO facilitates the reduction of light loss at short wavelengths compared to CdS, which transmits more photons to the absorber layer and improves the carrier collection capability. Benefits from high transparency of ZTO, favorable band alignment, wide bandgap, and reduced interface recombination, the champion device of Ag‐CZTSSe with ZTO buffer layer exhibited PCE of 11.8% which is the highest efficiency upto date among Cd‐free buffer layers. This work demonstrates the advantages of an eco‐friendly and highly transparent wide bandgap ZTO buffer layer and explores the significance of controlling the interface characteristics of CZTSSe/ZTO heterojunction.

## 4 Experimental Section

### Preparation of CZTS Solution

The precursor solution of CZTS was prepared by using the method reported in the literature.^[^ ^40^ ^]^ Briefly, CuCl, Zn(CH_3_COO)_2_.2H_2_O, SnCl_4_.5H_2_O, and SC(NH_2_)_2_, and Cu(CH_3_COO)_2_.2H_2_O, ZnCl_2_, SnCl_2_. 2H_2_O, SC(NH_2_)_2_ were added into 2‐methoxy ethanol, respectively. The solutions were kept at 60 °C for 2 h until a dark yellow color. The solution was diluted further to ½ of its initial concentration. In solution, Zn/Sn ratio was 1.2, and Cu/(Zn+Sn) ratio was 0.775. Ag‐based solution was formulated by mixing AgCl into thiourea and dimethyl sulfoxide (DMSO) solution.

### Fabrication of CZTSSe Film

Mo(molybdenum)‐coated soda‐lime glass (SLG) substrates were used to deposit CZTSSe thin film by spin coating with 4000 RPM for 30 s, afterward, the samples were subjected to heating at 280 °C for 2 min. The spin coating process was repeated several times for each device substrate to get desired thickness of CZTSSe film. Ag doping was achieved by adding AgCl solution to the precursor solution. For all samples, the spin‐coating step was repeated ten times. Then, the precursor thin films and selenium (Se) pellets were sealed inside the graphite box and heated at 565 °C for 15 min in a rapid thermal processing furnace under Argon (Ar_2_) flow. Utilizing solid selenium particles as Se source and the selenium amount was 80 mg. The selenization progress was carried out under a flowing Ar_2_ atmosphere at an atmospheric pressure. The furnace tube was evacuated four times by Ar_2_ before selenization. The electron transport layer was deposited after cooling down naturally.

### Fabrication of CdS Buffer Layer

The cadmium sulfide (CdS) buffer layer was deposited on selenized‐CZTS film via CBD. The CBD solution contained ammonia (14.8 m, 20 mL), thiourea (0.75 m, 20 mL), CdSO_4_ (0.015 m, 20 mL), and distilled water (140 mL). The complete process (CBD) was maintained for 9 min at 80 °C.

### Fabrication of Zinc–Tin‐Oxide Buffer Layer

ZTO films were deposited by the Fiji‐G2‐system (Nanotech). Diethyl‐zinc [Zn(C_2_H_5_)_2_], Tetrakis(dimethylamino)tin [Sn(N(CH_3_)_2_)_4_)], and H_2_O as a Zn, Sn, and oxygen source respectively. Zn(C_2_H_5_)_2_, and water were kept at room temperature while Sn(N(CH_3_)_2_)_4_ bottle was heated at 60 °C and the supply channel and substrate temperature were kept at 100 °C and 120 °C respectively. The ALD includes Zn/Sn: Argon:H_2_O: Argon along with a pulse time of 10 s/2 s:10 s:80 ms:10 s respectively. Four Zn to Sn sub‐cycle ratios (3/1, 4/1, 5/1, and 6/1 with a deposition rate of 0.07 nm per sub‐cycle were used.

### Fabrication of ITO and Ag

ITO thin films were directly deposited on the top of the buffer layer (CdS or ZTO) by the radio frequency (RF) magnetron technique. The process was carried out under the appropriate O_2_/Ar ratio (O_2_/Ar = 2%), at 100 W and 0.4 Pa pressure for 30 min. Ag metal electrodes were deposited under a high vacuum environment by thermal evaporation and the device area was measured to be 0.135 cm^2^.

### Characterization

The X‐ray diffraction (XRD) test was conducted using a Rigaku Ultima IV diffractometer equipped with a Cu K*α* radiation source. Renishaw inVia spectrometer with an excitation wavelength of 532 nm was used for Raman spectra. Scanning electron microscope (SEM) images were received using SEM‐SUPRA 55 system. Energy dispersive spectroscopy (EDX) (Bruker Quantax 200) was used for the chemical composition analysis of samples. Atomic force microscopy (AFM) images were obtained by NT‐MDT system using semi‐contact mode. CPD was measured by Kelvin probe force microscopy (KPFM) (Dimension Icon, Bruker) with SCM‐PIT probe under KPFM‐AM scan mode. Thermo Fischer Xi+ system was utilized to conduct X‐ray photoelectron spectroscopy (XPS). Transmission electron microscopy (TEM) analysis was performed by FEI Titan Cubed Themis G2 300 microscope. Ultraviolet photoelectron spectroscopy (UPS) was executed by PHI 5000 Versa Probe III with He I source (21.22 eV) under an applied negative bias of 9.0 V. The dark and illuminated *J–V* characteristics were measured by Keithley 2400 and Zolix SS150, and standard test condition (AM 1.5) was used to calibrate the solar simulator. External quantum efficiency (EQE) data were obtained using Zolix solar cell EQE/IPCE system with calibrated Si and InGaAs as references. Electrochemical impedance spectroscopy (EIS) measurement was taken using CHI66OE electrochemical workstation. Keithley 4200A‐SCS system was used for *C–V* and DLCP tests. Deep‐level transient spectroscopy (DLTS) was monitored by FT1030 HERA‐DLTS system.

## Conflict of Interest

The authors declare no conflict of interest.

## Supporting information

Supporting Information

## Data Availability

The data that support the findings of this study are available from the corresponding author upon reasonable request.
