# Supplementary material for: Cadmium‐Free Kesterite Thin‐Film Solar Cells with High Efficiency Approaching 12%
Source: Adv Sci (Weinh). 2023 Jun 30;10(26):2302869. doi: 10.1002/advs.202302869 (PMC10502672; doi:10.1002/advs.202302869)
Supplement: Supplementary file 1 — Supporting Information [file ADVS-10-2302869-s001.pdf]

ADVANCED SCIENCE
Open Access

Supporting Information

for *Adv. Sci.*, DOI 10.1002/advs.202302869

Cadmium-Free Kesterite Thin-Film Solar Cells with High Efficiency Approaching 12%

Nafees Ahmad, Yunhai Zhao, Fan Ye, Jun Zhao, Shuo Chen, Zhuanghao Zheng, Ping Fan, Chang Yan, Yingfen Li, Zhenghua Su, Xianghua Zhang and Guangxing Liang**

Supporting Information

Cadmium-free Kesterite Thin-Film Solar Cells with High Efficiency approaching 12%

Nafees Ahmad, Yunhai Zhao, Fan Ye, Jun Zhao, Shuo Chen, Zhuanghao Zheng, Ping Fan, Chang Yan, Yingfen Li, Zhenghua Su, Xianghua Zhang, Guangxing Liang**

Table S1. Photovoltaic parameters of CdS and ZTO with different (Zn: Sn) ratios and ALD cycles.

Zn: Sn ratio	ETL	Voc [V]	Jsc [mA/cm^2]	FF [%]	PCE [%]
	CdS	0.49	34.8	62.9	10.7
3:1	ZTO (12 cycles)	0.48	34.5	58.1	9.6
	ZTO (25 cycles)	0.48	36.1	63.0	10.8
	ZTO (50 cycles)	0.47	32.5	61.1	9.2
	ZTO (75 cycles)	0.47	34.1	53.0	8.4
4:1	ZTO (12 cycles)	0.47	33.0	61.5	9.5
	ZTO (25 cycles)	0.49	35.2	65.5	11.2
	ZTO (50 cycles)	0.46	35.7	61.2	9.9
	ZTO (75 cycles)	0.46	31.8	55.4	8.1
5:1	ZTO (12 cycles)	0.46	35.1	61.0	9.8
	ZTO (25 cycles)	0.49	36.2	66.5	11.8
	ZTO (50 cycles)	0.47	34.5	63.1	10.0
	ZTO (75cycles)	0.47	34.6	58.5	9.4
6:1	ZTO (12 cycles)	0.46	34.2	58.4	9.1
	ZTO (25 cycles)	0.49	35.6	64.1	11.0
	ZTO (50 cycles)	0.48	34.3	55.0	9.0
	ZTO (75 cycles)	0.47	34.0	53.7	8.4

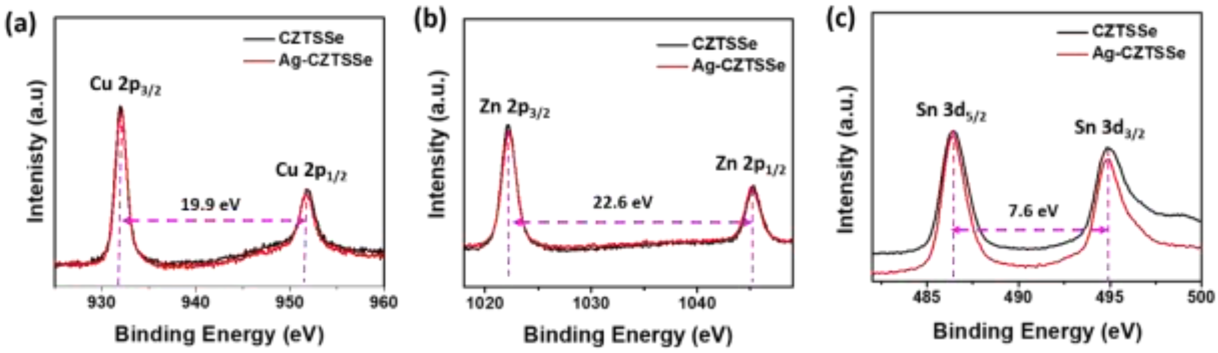

Figure S1. XPS spectra of (a) Cu 2p, (b) Zn 2p, (c) Sn 3d peaks of CZTSSe and Ag-CZTSSe thin films.

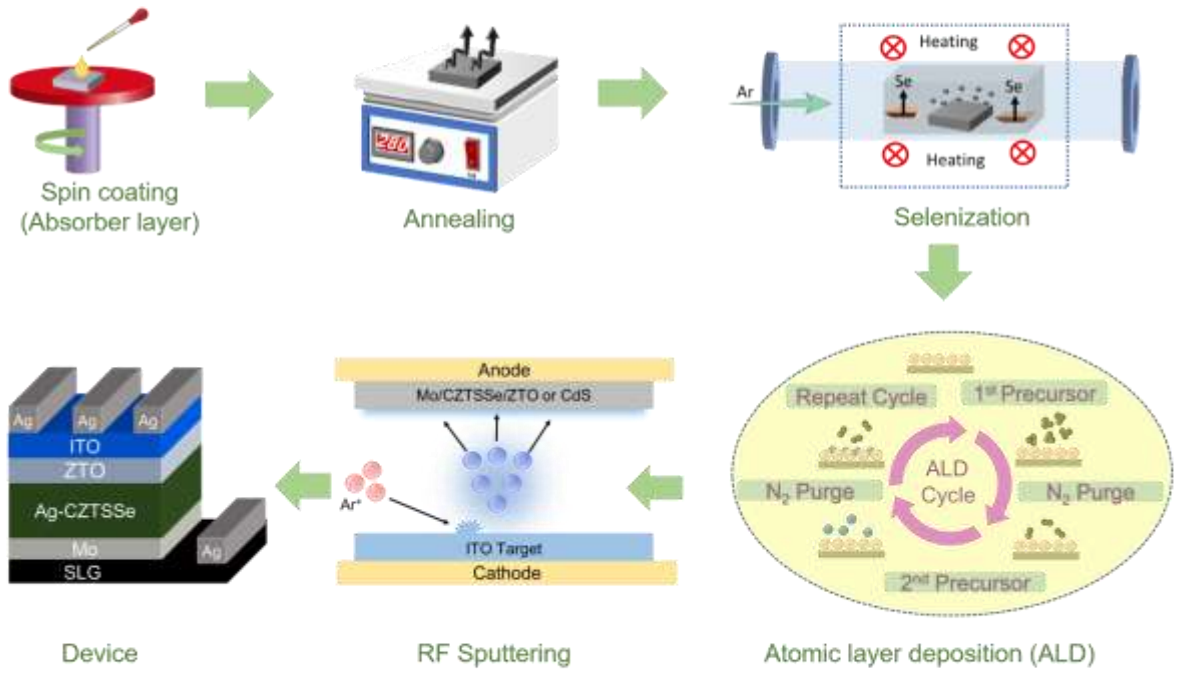

Figure S2. Schematic illustration of the preparation process of CZTSSe thin film solar cell.

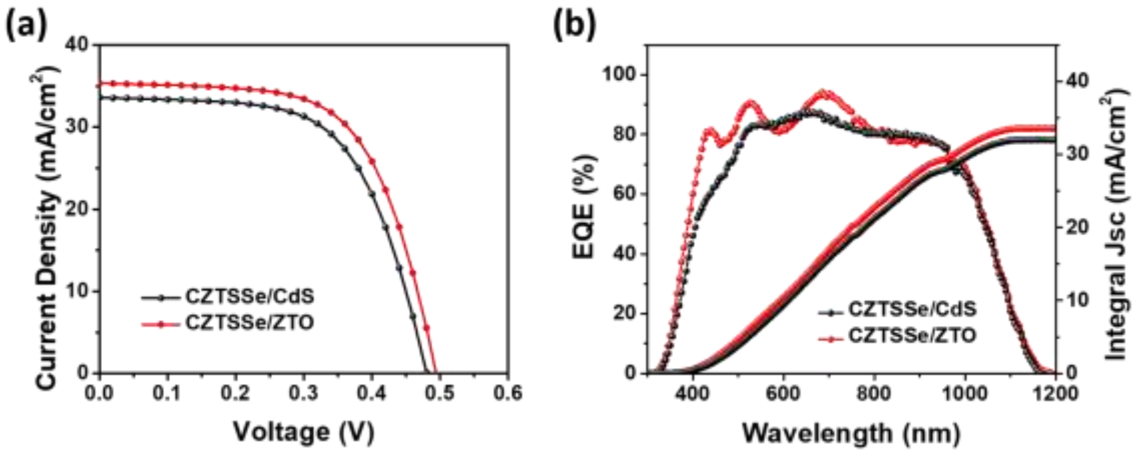

Figure S3. (a) J-V curve of CdS and ZTO based CZTSSe solar cells. (b) EQE spectra of the corresponding devices.

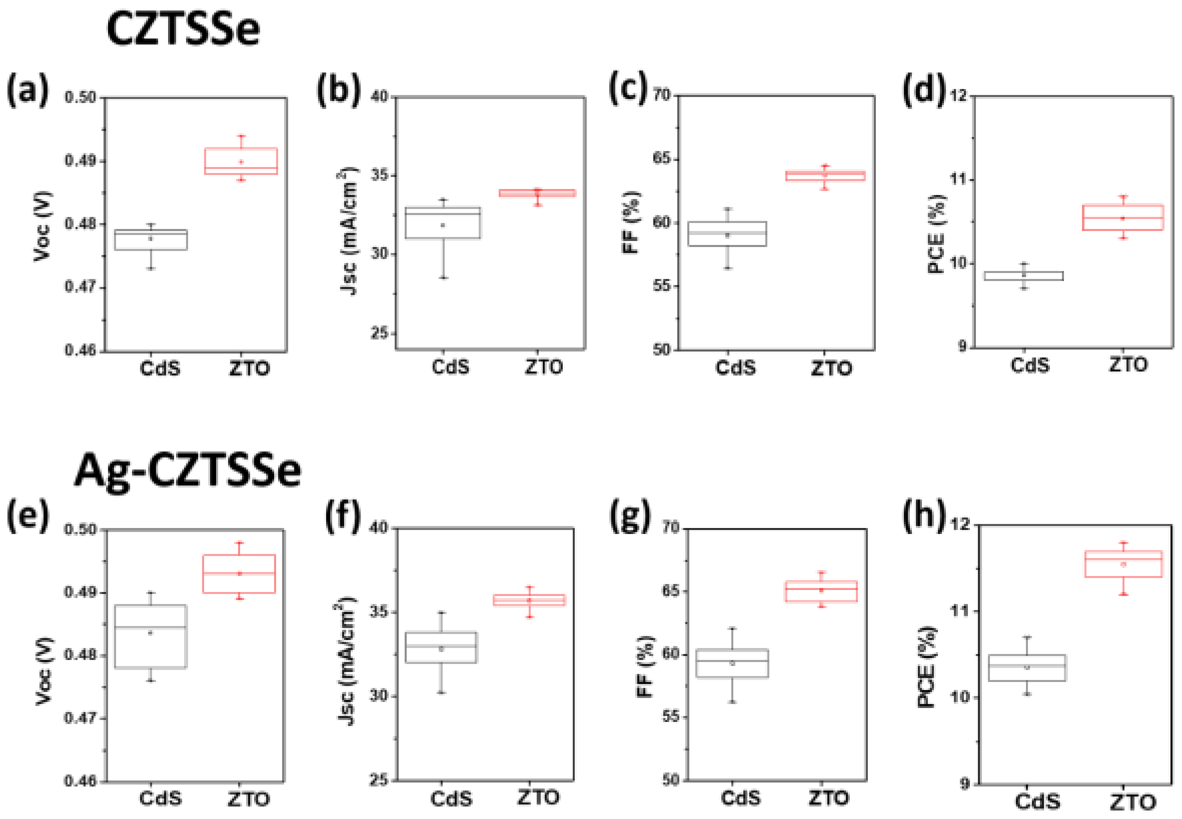

Figure S4. Statistical box diagrams of photovoltaic parameters including (a, e) Voc, (b, f) Jsc, (c, g) FF, and (d-h) PCE of CZTSSe and Ag-CZTSSe thin film solar cell with CdS and ZTO buffer layer (10 cells were selected for each sample).

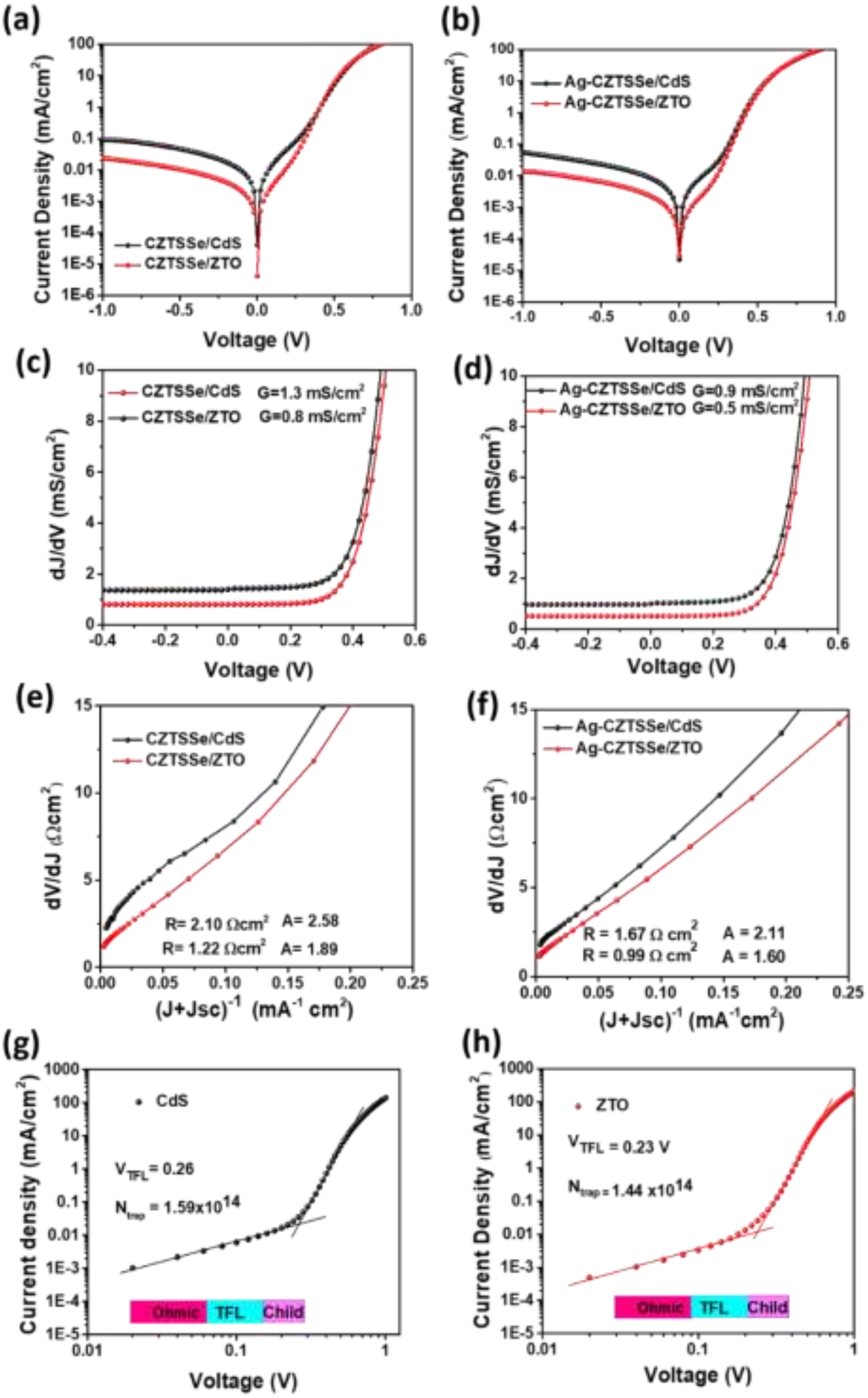

Figure S5. Electrical behavior of CZTSSe and Ag-CZTSSe thin film with CdS and ZTO buffer layer. (a-b) Dark J-V, (c-d) shunt conductance G, (e-f) Series resistance R and ideality factor A, (g-h) Logarithmic J-V curves of corresponding devices showing ohmic, TFL, and Child region.

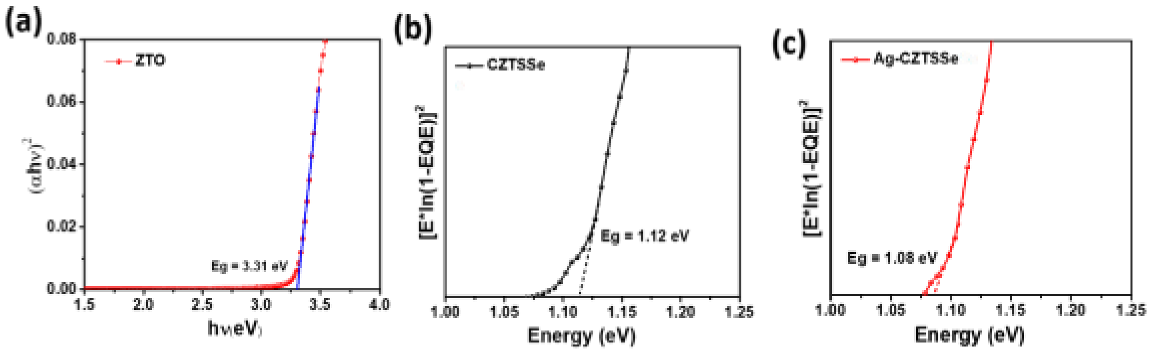

Figure S6. (a) Tauc plot for calculation of band-gap of ZTO, (b) Band gap of CZTSSe, (c) and Ag-CZTSSe thin films calculated from EQE data.

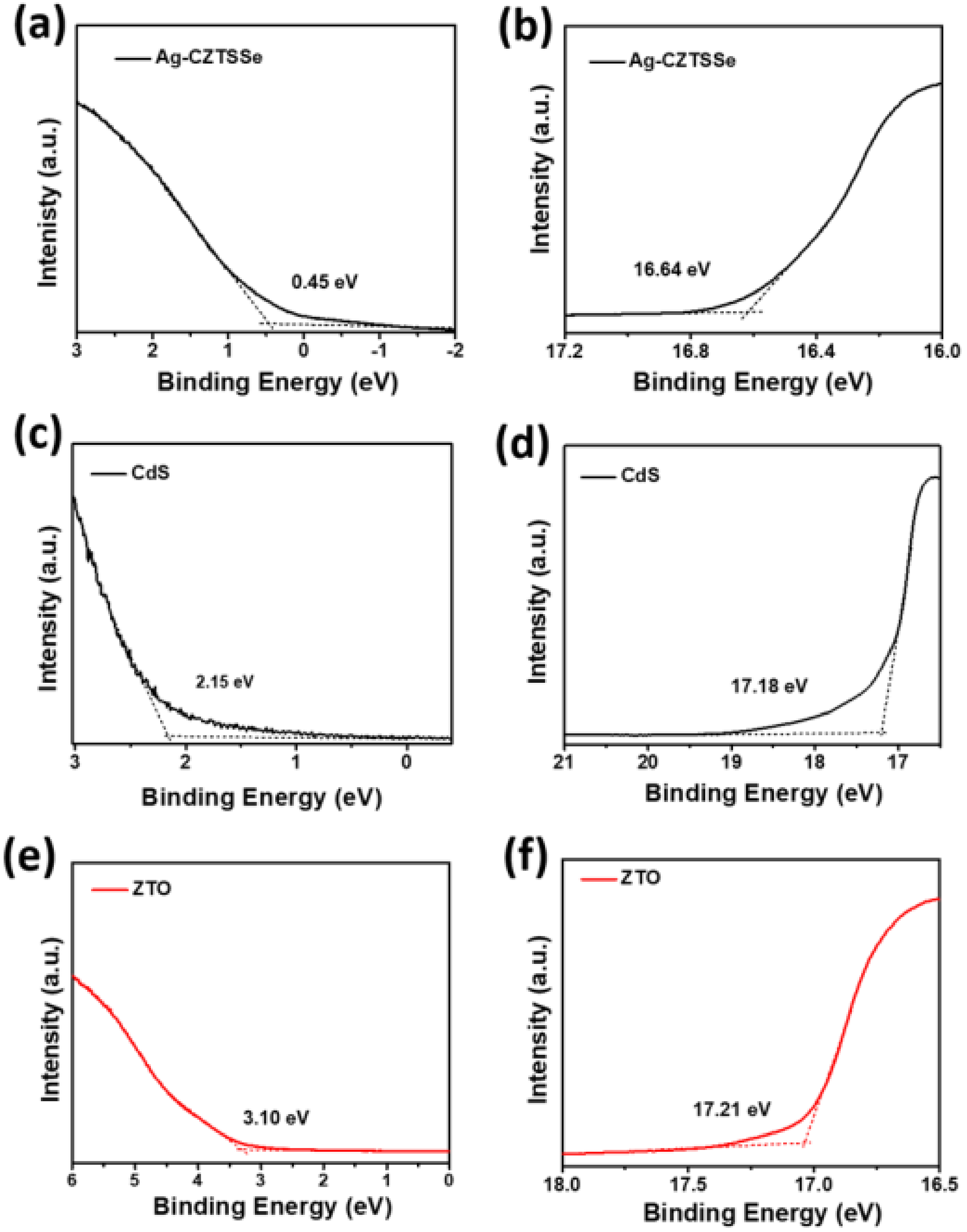

Figure S7. The energy of the Fermi level relative to the valence band ($E_{Fermi-VB}$) and the secondary electron cut-off edge ($E_{cut-off}$) derived from UPS for; (a-b) Ag-CZTSSe, (c-d) CdS, and (e-f) ZTO thin film.

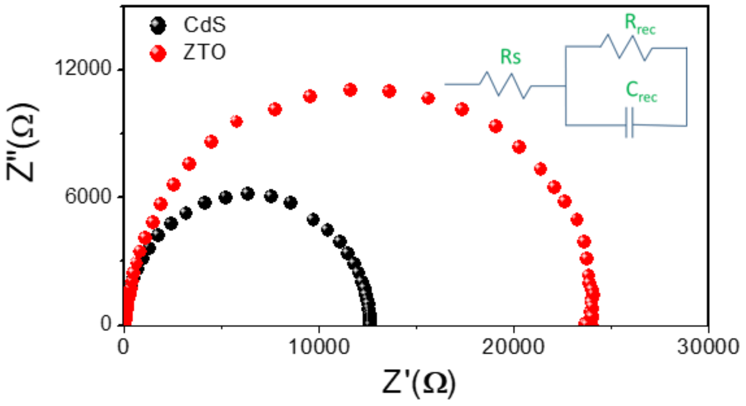

Figure S8. EIS Nyquist plots of Ag-CZTSSe-based thin film with CdS and ZTO buffer layer. Inset is the equivalent circuit diagram.
